# An eight-year follow-up national study of medical school and general hospital ethics committees in Japan

**DOI:** 10.1186/1472-6939-8-8

**Published:** 2007-06-29

**Authors:** Akira Akabayashi, Brian T Slingsby, Noriko Nagao, Ichiro Kai, Hajime Sato

**Affiliations:** 1 Department of Biomedical Ethics, Graduate School of Medicine University of Tokyo University of Tokyo 7-3-1 Hongo, Bunkyo-ku, Tokyo 113-0033, Japan; 2 Department of Social Gerontology, Graduate School of Medicine University of Tokyo University of Tokyo 7-3-1 Hongo, Bunkyo-ku, Tokyo 113-0033, Japan; 3 Department of Public Health, Graduate School of Medicine University of Tokyo University of Tokyo 7-3-1 Hongo, Bunkyo-ku, Tokyo 113-0033, Japan

## Abstract

**Background:**

Ethics committees and their system of research protocol peer-review are currently used worldwide. To ensure an international standard for research ethics and safety, however, data is needed on the quality and function of each nation's ethics committees. The purpose of this study was to describe the characteristics and developments of ethics committees established at medical schools and general hospitals in Japan.

**Methods:**

This study consisted of four national surveys sent twice over a period of eight years to two separate samples. The first target was the ethics committees of all 80 medical schools and the second target was all general hospitals with over 300 beds in Japan (n = 1457 in 1996 and n = 1491 in 2002). Instruments contained four sections: (1) committee structure, (2) frequency of annual meetings, (3) committee function, and (4) existence of a set of guidelines for the refusal of blood transfusion by Jehovah's Witnesses.

**Results:**

Committee structure was overall interdisciplinary. Frequency of annual meetings increased significantly for both medical school and hospital ethics committees over the eight years. The primary activities for medical school and hospital ethics committees were research protocol reviews and policy making. Results also showed a significant increase in the use of ethical guidelines, particularly those related to the refusal of blood transfusion by Jehovah's Witnesses, among both medical school and hospital ethics committees.

**Conclusion:**

Overall findings indicated a greater recognized degree of responsibilities and an increase in workload for Japanese ethics committees.

## Background

Ethics committees (EC) and the system of research protocol peer-review began in the United States (US) [1,2]. The Declaration of Helsinki requires that all biomedical research involving human participants, including research on identifiable human material or data, should be approved by an ethical review committee [3,4]. Today, ECs and their systems of peer-review for research protocol are used worldwide [5-16].

Nowadays ECs in many countries review and consider ethical issues pertaining to clinical care and institutional policy. In fact, clinical ECs often function independently from research ethics committees [17,18]. For instance, in the United Kingdom (UK), clinical ECs largely function for ethics support and advice for healthcare providers [17-20]. In the US, institutional or hospital ECs (HECs) carry out ethics consultation and other clinically relevant activities [1].

Japanese ECs differ in system, however, from those in the US and UK. The first medical school in Japan to establish an EC was Tokushima University School of Medicine in 1982. Ten years later, in 1992, all 80 medical schools in Japan had voluntarily established an EC without any governmental regulation. This same trend emerged among general hospitals as well. The percentage of hospitals with over 300 beds that maintained an EC increased from 24.6% in 1996 to 52.0% in 2002 according to the Ministry of Health, Labour and Welfare [21,22].

In Japan, the term for ethics committees (*rinri-iinkai*) is often translated as an IRB, but this is quite misleading. At medical schools and the majority of general hospitals, there are actually two types of ECs [23]: an EC that reviews and monitors drug clinical trials called a *chiken-shinsa-iinkai *(clinical trial review committee), and an EC that reviews protocols from researchers affiliated with the institution called a *rinri-iinkai *(ethics committee). Clinical trial review committees are regulated by the Ministry of Health, Labor and Welfare and function in accordance with the Pharmaceutical Law and the Guidelines for Good Clinical Practice (GCP)[23]. Accordingly, their structure and management are strictly regulated. Hospitals and universities involved with clinical trials for commercial products (i.e., pharmaceuticals) are thus required by the GCP to maintain a clinical trial review committee. Conversely, ECs not involved with clinical trials for commercial products are primarily self-governing bodies established by each institution and are not government regulated. In general, medical school ECs are more involved with research protocol review than their counterpart in hospitals; they also play a larger role as a leader in policy making when compared to hospital ECs.

Today, concerns over the quality and function of ECs are increasing worldwide. Numerous studies have examined the activities and quality of ethical review of ECs among Westernized countries included Australia, Belgium, Canada, Israel, Italy, France, Germany, Netherlands, New Zealand, Spain, Sweden, Switzerland, the UK and the US [5-8,11-14,16-18]. Many of these studies have provided descriptive data on EC functions and structure [9-12,16]. Yet, despite the wealth of literature from Westernized countries, there persists a limited amount of research from Asia [10,15]. The purpose of this survey study was to describe the characteristics and developments of ECs established at medical schools and general hospitals in Japan. In general, medical school and hospital ECs are combined ethics committees taking on the roles of both clinical and research ethics committees. Japanese ECs are regulated independently by each facility as opposed to the British system in which ECs are established based on area (LREC: local research ethics committee), and accredited according to the rules formulated by the UK Ethics Committee Authority [24]. We excluded clinical trial review committees from our sample given their differences in structure, function and regulations when compared to regular ECs. This study included four national surveys conducted twice over a period of eight years.

## Methods

The study consisted of four national surveys sent to two separate samples. The first target was the ECs of all 80 medical schools and the second target was all general hospitals with over 300 beds in Japan. In July 1995 and August 2002, we mailed a self-administered instrument to the EC at all 80 medical schools. By 1995, all Japanese medical schools had established an EC. Concurrently, we mailed a self-administered instrument addressed to the Chief of Staff at all general hospitals with over 300 beds in April 1996 (n = 1457) and March 2002 (n = 1491). This sample of general hospitals did not include university hospitals.

### Instrument

Each instrument contained four sections: (1) committee structure; (2) frequency of annual meetings; (3) committee function; (4) existence of ethical guidelines for the refusal of blood transfusion by Jehovah's Witnesses. We chose to use number four because the existence of ethical guidelines for dealing with Jehovah's Witnesses in Japan can serve as a possible indicator of EC functionality. Two additional sections were included in instruments developed for medical school ECs that pertained to awareness of the binding power of committee decisions and awareness of recent changes in responsibility.

### Analysis

All data were edited and analyzed using SPSS Windows Version 11.0 to determine frequency of responses for each category. To measure the strength of association between variables, tests of significance, such as the *t *test and Chi Squared test, were calculated.

### Ethical considerations

The study was approved by the University of Tokyo Graduate School of Medicine Research Ethics Committee. All instruments were mailed with a letter explaining the objectives of the study, protection of personal information, and how all responses were voluntary.

## Results

From medical schools, 80 instruments were returned in 1995 (response rate 100%) and 62 were returned in 2002 (response rate 77.5%). From hospitals, 743 instruments were returned in 1996 (response rate 51.0%) and 464 instruments were returned in 2002 (response rate 31.1%). The results of the 1996 survey of general hospital ECs were partly presented previously in Japanese [25].

### Respondent attributes (table 1) and ratio of established ECs (table 2)

**Table 1:** Respondents to the medical school survey

	1995		2002	
	N = 80	%	N = 62	%
**Sex**				
Male	80	100	60	96.8
Female	0	0.0	2	3.2
**Age**				
30's	2	2.5	0	0.0
40's	5	6.3	4	6.5
50's	15	18.8	17	27.4
60's	54	67.5	40	64.5
70's	1	1.3	1	1.6
NA	3	3.8	0	0.0
**Committee Position**				
Committee chair	67	83.8	52	83.8
Committee co-chair	3	3.8	4	6.5
Member	3	3.8	6	9.7
Other	5	6.3	0	0.0
NA	2	2.5	0	0.0
**University Type**				
National University	32	40	25	40.3
National College	19	23.8	15	24.2
Private University	11	13.8	5	8.1
Private College	18	22.5	17	27.4

**Table 2:** Respondents to the general hospital survey

	1996		2002	
	N = 743	%	N = 464	%
**Sex**				
Male	624	84.0	418	90.1
Female	7	0.9	0	0.0
NA	112	15.1	46	9.9
**Age**				
20's	0	0.0	1	0.2
30's	17	2.3	3	0.6
40's	85	11.4	44	9.5
50's	255	34.3	156	33.6
60's	325	43.7	233	50.2
70's	37	5.0	12	2.6
NA	24	3.2	15	3.2
**Hospital Position**				
Chief of Staff	536	72.1	364	78.4
Co-chief of Staff	110	14.8	57	12.3
Physician	46	6.2	23	5.0
Administrator	23	3.1	10	2.2
Other	6	0.8	2	0.4
NA	22	3.0	8	1.7
**Hospital Type**				
National	97	13.1	46	9.9
Public	326	43.9	270	58.1
Private	265	35.7	140	30.2
Other	35	4.7	0	0.0
NA	20	2.7	8	1.7

**Figure 1:**
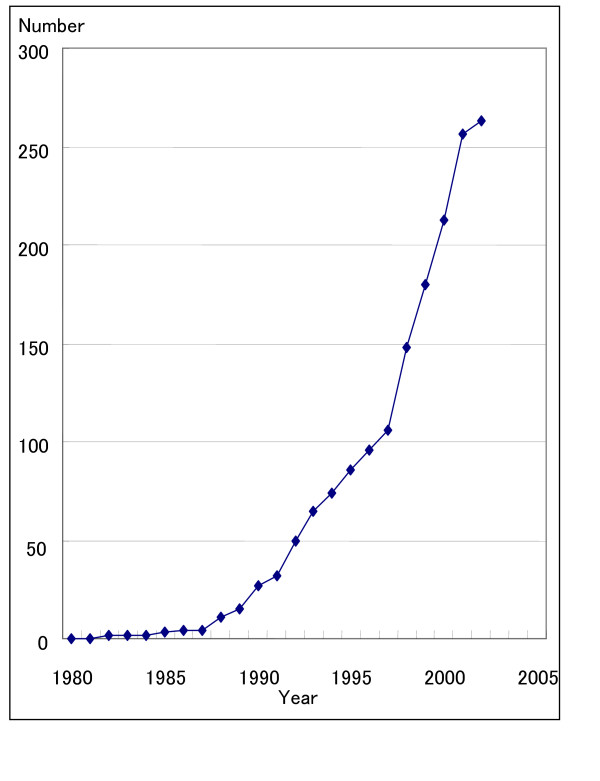
The Number of Ethics Committees Established at General Hospitals in Japan. *Data were obtained from 2002 hospital survey.

Table 1 and Table 2 report the attributes of the respondents. In 1995, all medical schools (n = 80) had an established ECs. Conversely, only 181 (24.4%) of general hospitals in this study responded that they had established an EC in 1996[15] (result from reference 15), which increased significantly to 270 (58.2%) by 2002 (Figure 1: Number of Ethics Committees Established at General Hospitals in Japan).

### Structure of ethics committees at medical schools (table 3) and hospitals (table 4)

**Table 3:** Characteristics of ethics committees

	Medical School	
Characteristics	Mean ± SD	Range
**Total members**	10.4 ± 2.6	6–22
External members (outside of medical school but within same college/university)	1.3 ± 1.3	0–6
External members (outside of same college/university)	1.4 ± 1.1	0–5
Female members	0.6 ± 0.9	0–6
**Discipline of EC members**		
Basic medicine	2.7 ± 1.1	1–8
Clinical medicine	4.0 ± 1.6	1–8
Other medicine	0.7 ± 1.1	0–4
Nursing	0.3 ± 0.6	0–3
Other healthcare field	0.1 ± 0.4	0–3
Law	1.0 ± 0.6	0–3
Philosophy/Ethics	0.5 ± 0.6	0–3
Other Humanities and/or Social Sciences	0.4 ± 0.7	0–3
Administrative	0.2 ± 0.6	0–4
Other	0.5 ± 0.9	0–4

**Table 4:** Characteristics of ethics committees

	General Hospital	
Characteristics	Mean ± SD	Range
Total members	10.3 ± 4.9	4–27
External members	1.2 ± 1.4	0–8
Female members	1.3 ± 1.3	0–8
**Discipline**		
Chief of staff	0.7 ± 0.04	0–1
Co-chief of staff	1.4 ± 0.9	0–4
Physician	4.1 ± 4.3	0–18
Nurse	1.2 ± 1.0	0–7
Co-medical staff	0.5 ± 0.9	0–5
Administrator	0.5 ± 1.0	0–4
Attorney/Consultant	0.4 ± 0.6	0–3
Philosopher/Ethicist	0.3 ± 0.6	0–4
Other	0.4 ± 0.9	0–6

Table 3 and Table 4 each report the structure of ECs at responding institutions. All data was collected during the initial surveys. The overall ratio of male to female committee members was 94:6 among medical school ECs and 87:13 among hospital ECs. Medical school ECs consisted of an average of more than one member from outside of the medical school, yet within the same college/university, as well as from altogether outside of the college/university. Hospital ECs also consisted of an average of more than one member who was unaffiliated with the facility. In general, the discipline of EC members varied. Medical doctors and healthcare professionals comprised the majority, but medical school ECs included approximately one legal adviser. Professionals of ethics and other humanities were also included.

### Process of ethics committees

Findings indicated an increase in workload among ECs. Medical school ECs met an average of 3.6 (SD:3.6) times in the year prior to the survey in 1995 and 7.7 (SD:4.6) times in the year prior to the survey in 2002. Hospital ECs met an average of 2.4 (SD:3.2) times in the year prior to the survey in 1996 and 3.1 (SD: 3.3) times in the year prior to the survey in 2002. In both cases, the frequency of meetings per year showed a significant increase (p < 0.01). Number of cases discussed per year for medical school ECs increased significantly from 5.7 (SD:5.7) cases in 1995 to 51.2 (SD:52.0) cases in 2002 (p < 0.01).

### Activities of ethics committees at medical schools (table 5) and hospitals (table 6)

**Table 5:** Activities at medical school ECs 1995 (n = 80)

	(%)
Review of clinical research related to treatment with patients as subjects	74(92.5)
Review of clinical research unrelated to treatment with patients as subjects	62(77.5)
Review of clinical research with non-patients as subjects	58(72.5)
Review of basic research with animals as subjects	16(20.0)
Issuing of a committee approval certificate for the editorial board of an academic journal	27(33.8)
Policy-making	66(82.5)
Consultation	14(17.5)
Education	17(21.3)

**Table 6:** Activities at hospital ECs 1996 (n = 180)

	(%)
Review of clinical research related to treatment with patients as subjects	127(70.6)
Review of clinical research unrelated to treatment with patients as subjects	52(28.9)
Review of clinical research with non-patients as subjects	34(18.9)
Review of basic research with animals as subjects	9(0.5)
Issuing of a committee approval certificate for the editorial board of an academic journal	19(10.6)
Policy making	99(55.5)
Consultation	58(32.2)
Education	30(16.7)

The activities of medical school and hospital ethics committees are reported in Tables 5 and 6. Answers were multiple-choice. An average of 85.0% of medical school ECs responded that their everyday activities consisted of reviews of protocols for patient-targeted clinical research, regardless of whether they were related to medical treatment. Over 80% of medical school ECs were also involved in policy making, defined here as the establishing of regulations and guidelines for a certain treatment or research subject. Conversely, hospital ECs were primarily involved in the ethical review of protocols for patient-targeted clinical research directly related to medical treatment (70.6%). Compared to medical school ECs, fewer hospital ECs were involved in the review of protocols for patient-targeted clinical research unrelated to medical treatment (28.9%). Hospital ECs were also considerably involved in policy making (55.5%). A comparison of medical school and hospital ECs found that medical school ECs were more involved in the issuing of certificates intended for the editorial board of an academic journal than hospital ECs (33.8%:10.6%), and that hospitals ECs were more involved in ethics consultation (17.5%:32.2%).

### Use of ethical guidelines

Instruments included a question on whether ECs had implemented an ethical guideline on the refusal of blood transfusion based on religious reasons (Jehovah's Witnesses). Among medical schools ECs in 1995, 31 (38.8%) responded yes to this question; 29 (36.3%) replied no; 13 (16.3%) responded that they were still considering it; five (6.3%) replied other and two (2.5%) did not reply. Conversely, in 2002, 35 (56.5%) medical schools replied yes to this question; 16 (25.8%) replied no; six (9.7%) replied that they were still considering it; three (4.8%) replied other and two (3.2%) did not reply. A significant increase was found in the use of an ethical guideline among medical school ECs (χ^2 ^= 4.6, p < 0.05). Again, among hospitals ECs, 142 (19.1%) in 1996 replied yes to this question; 533 (71.7%) replied no; 57 (7.7%) replied that they were still considering it and 11 (1.5%) did not reply (result from reference 22). Conversely, in 2002, 159 (34.3%) replied yes; 265 (57.1%) replied no; 23 (5.0%) replied that they were still considering it and 17 (3.7%) did not reply. A significant increase was found in the use of an ethical guideline among hospital ECs (χ^2 ^= 35.5, p < 0.01).

### Awareness of binding power and legal liability

Instruments targeted to medical school ECs included a question on how much binding power and legal liability they considered their decisions to carry. A total of 51 (63.8%) ECs in 1995 compared to 20 (32.3%) in 2002 believed that, "Since decisions are mere advice and have no binding power, ECs cannot take disciplinary action and have no legal liability." Seventeen (21.3%) ECs in 1995 compared to 28 (45.2%) in 2002 believed that, "Although decisions carry binding power and ECs can take disciplinary action, it is the university or university hospital, not the EC itself, that has legal liability." Three (3.8%) ECs in 1995 compared to 7 (11.3%) in 2002 believed that, "Since decisions carry binding power and ECs can take disciplinary action, the EC has legal co-liability with the university or university hospital." Nine (11.3%) ECs in 1995 and 7 (11.3%) in 2002 answered "don't know or other." These results show a significant increase in awareness of decision binding power and legal liability among medical school ECs (χ^2 ^= 15.8, p < 0.01).

### Recent trends

Instruments targeted to medical school ECs in 2002 also included a question on whether the topic of reviews had diversified in recent years. Forty (64.5%) ECs replied "much more diversified" and 19 (30.6%) answered "slightly more diversified." Only 2 (3.2%) replied "no change"; 1 (1.6%) answered "slightly less diversified," and none replied "much less diversified." In regards to an additional question that asked whether responsibilities have increased in recent years, 40 (64.5%) ECs replied "much more" and 18 (29.0%) answered "slightly more." Four (6.5%) ECs replied "no change" and none answered "much less" or "slightly less."

## Discussion

The goal of this study was to gain an overview of the characteristics and developments of ECs established at medical schools and general hospitals in Japan with a series of four national surveys conducted over a period of eight years. The descriptive results intimate a gradual growth in number of ECs, an increase in frequency of annual meetings and number of reviews, an increase in the use of ethical guidelines, and a greater recognized degree of responsibilities for Japanese ECs.

According to reports by the Ministry of Health, Welfare and Labor, the number of ECs (*rinri-iinkai*) in general hospitals with over 300 beds increased from 24.6% in 1996 to 52.0% in 2002 [21,22]. One possible factor related to this increase in number of ECs among general hospitals in Japan is the recent introduction of a system of evaluation [26]. In fact, it was in 1998 when the Japan Council for Quality Healthcare (JCQH), a non-governmental agency that evaluates hospitals, added the category of EC in their evaluation instrument. Again, yet another factor related to this increase is the recent succession of governmental ethical guidelines. Around the turn of the century, a series of statutory guidelines, in all seven guidelines, strongly "recommended" that institutions establish an EC [23]. For instance, the Ethical Guideline for Human Genome and Gene Analysis Research and the Ethical Guideline for Epidemiological Research mandated that all related-research need to undergo EC review. Accordingly, this change in policy may also have had an impact on the increase of ECs and on their increase in responsibility and number of reviews. This study was carried out before and after the addition of the evaluative category of ECs by the JCQH in 1998, as well as before and after the succession of governmental ethical guidelines around 2000. Study findings thus reflect the impact of these events.

The system of ECs in Japan has two unique and noteworthy characteristics: (1) medical schools and the majority of hospitals have established their EC voluntarily without any governmental regulation, and (2) ECs play the roles of both IRB and HEC, as defined in the US. Now even though medical schools and hospitals voluntarily set up their EC, their structure is largely similar. They all include members who are external, of both sexes, and from other fields than medicine. This standardization may largely be a product of the Liaison Society for Ethics Committees of Medical Schools [27], an association set up in 1988 among all medical school ECs to exchange information and communicate. Presently, the association meets two times a year to discuss relevant issues to the further development and needs of ECs. To our knowledge, no other nation has such an association that functions as a national alliance of medical school ECs. Incidentally, another factor related to the apparent homogeneity in structure among ECs may be the succession of ethical guidelines issued by the government around the turn of the century.

A second significant characteristic of ECs in Japan is that they are not only involved with review of research protocols, like IRBs in the US, but are also in charge of policy making, education, and consultation, similar to HEC in the US context. The reason for this double-role playing among Japanese ECs is because the GCP originally assigned all drug clinical trials to clinical trial review committees (*chiken-shinsa-iinkai*), and left the remaining responsibilities to ECs (*rinri-iinkai*). Today, the primary role for medical school and hospital ECs is research protocol review and policy making.

Study findings also showed a significant increase in the use of ethical guidelines among both medical school and hospital ECs. However, we also found a difference between medical school and hospital ECs. In 1995–6, 38.5% of medical school ECs compared to 19.1% of hospital ECs used an ethical guideline for blood transfusion by Jehovah's Witnesses. Again this difference was seen in 2002 with 56.5% of medical schools ECs using an ethical guideline compared to 34.3% of hospital ECs. We surmise that this difference is because medical school ECs tend to be more active than hospital ECs in developing policy. One possible explanation for this disparity is that medical school ECs generally function as a leader in developing ethical policy in Japan. Looking deeper, this may be related to the hierarchy of Japan's medical world in which universities hold the mainstay of power.

Overall findings showed a greater degree of responsibilities and an increase workload for Japanese ECs. The vast majority of ECs indicated that their responsibilities have increased "much more" in recent years. This is related to several factors: ECs having to function both as IRB and HEC, the rush of ethical guidelines, and an overall rise in social awareness regarding bioethical issues [28]. Whether a standard of quality is being maintained among Japanese ECs given the increase in workload and responsibilities is a topic of future study and discussion. To ensure a consistent standard of quality of review and function among ECs in Japan, researchers and policy makers need to consider the possibilities of a central IRB, the introduction of a system of registration for ECs, increased legal binding power for committee decisions and the further development of ethics consultation.

## Limitations

This study is subject to several limitations. First, the response rate among general hospitals was low, yet since the ratio of established hospital ECs in this study was similar to that officially reported by the Ministry of Health, Labor and Welfare, this study's sample is thought to be representative. Second, we cannot determine whether nonrespondents would have answered questions differently. Third, we surveyed only one member of each institution, whose assessments and judgments may differ from those of the committee as a whole. Fourth, the use of an ethical guideline for dealing with Jehovah's Witnesses may not serve as a valid index for adequate functioning of a committee. Further research is needed in order to develop a scale to assess the efficacy of functioning of a committee. Lastly, respondents will at best provide approximate answers to activity distributions and the like. Although these responses are subject to errors of recall and estimation, they are adequate in providing a current view of ECs in Japan.

## Conclusion

Ethics committees play an essential role in research and healthcare by aiming to ensure the safety of patients and research participants. While the system of ECs in Japan was largely imported from the West, its history of development and systems of management are unique. Ethics committees and the system of research protocol peer-review continue to spread worldwide among nations that differ in culture, healthcare system and governmental structure. This is a positive trend, yet to better ensure a common international standard among all nations worldwide, further data and research are needed on each nation's system of EC.

## Competing interests

The author(s) declare that they have no competing interests.

## Authors' contributions

AA conceived and designed the study, and collected the data. BTS conducted data analysis and contributed to the writing of the paper. NN assisted with the data analysis. IK and HS supervised data collection and analysis. All authors read and approved the final manuscript.

## Pre-publication history

The pre-publication history for this paper can be accessed here:


